# Integrative Transcriptomic and Metabolic Analyses Reveal That Flavonoid Biosynthesis Is the Key Pathway Regulating Pigment Deposition in Naturally Brown Cotton Fibers

**DOI:** 10.3390/plants13152028

**Published:** 2024-07-24

**Authors:** Shandang Shi, Rui Tang, Xiaoyun Hao, Shouwu Tang, Wengang Chen, Chao Jiang, Mengqian Long, Kailu Chen, Xiangxiang Hu, Quanliang Xie, Shuangquan Xie, Zhuang Meng, Asigul Ismayil, Xiang Jin, Fei Wang, Haifeng Liu, Hongbin Li

**Affiliations:** 1 Key Laboratory of Xinjiang Phytomedicine Resource and Utilization of Ministry of Education, Key Laboratory of Oasis Town and Mountain-Basin System Ecology of Bingtuan, College of Life Sciences, Shihezi University, Shihezi 832000, China; 2 China Colored-Cotton (Group) Co., Ltd., Urumqi 830023, China; 3 Ministry of Education Key Laboratory for Ecology of Tropical Islands, Key Laboratory of Tropical Animal and Plant Ecology of Hainan Province, College of Life Sciences, Hainan Normal University, Haikou 571158, China; 4 Rural Energy and Environment Workstation of Yili State, Yining 835000, China

**Keywords:** naturally colored cotton, brown cotton fiber, pigment deposition, transcriptome, metabolome, flavonoid biosynthesis

## Abstract

Brown cotton is a major cultivar of naturally colored cotton, and brown cotton fibers (BCFs) are widely utilized as raw materials for textile industry production due to their advantages of being green and dyeing-pollution-free. However, the mechanisms underlying the pigmentation in fibers are still poorly understood, which significantly limits their extensive applications in related fields. In this study, we conducted a multidimensional comparative analysis of the transcriptomes and metabolomes between brown and white fibers at different developmental periods to identify the key genes and pathways regulating the pigment deposition. The transcriptomic results indicated that the pathways of flavonoid biosynthesis and phenylpropanoid biosynthesis were significantly enriched regulatory pathways, especially in the late development periods of fiber pigmentation; furthermore, the genes distributed in the pathways of *PAL*, *CHS*, *F3H*, *DFR*, *ANR*, and *UFGT* were identified as significantly up-regulated genes. The metabolic results showed that six metabolites, namely (−)-Epigallocatechin, Apiin, Cyanidin-3-O-glucoside, Gallocatechin, Myricetin, and Poncirin, were significantly accumulated in brown fibers but not in white fibers. Integrative analysis of the transcriptomic and metabolomic data demonstrated a possible regulatory network potentially regulating the pigment deposition, in which three MYB transcription factors promote the expression levels of flavonoid biosynthesis genes, thereby inducing the content increase in (−)-Epigallocatechin, Cyanidin-3-O-glucoside, Gallocatechin, and Myricetin in BCFs. Our findings provide new insights into the pigment deposition mechanism in BCFs and offer references for genetic engineering and breeding of colored cotton materials.

## 1. Introduction

Cotton fibers are the largest natural textile materials and hold significant economic importance [1]. In fabric processing and manufacturing, dyes are commonly used to enhance the aesthetics and functionality of textiles [2,3]. However, the associated environmental pollution, resource consumption, and health risks cannot be ignored [4]. Naturally colored cotton (NCC), a variety of upland cotton that contains natural pigments in fibers, does not require chemical bleaching and dyeing during processing, thereby reducing environmental pollution and health hazards [5]. Therefore, despite the disadvantages of relatively poor quality, colors being limited to brown and green, and unstable pigment inheritance, NCC is still consistently considered an eco-friendly cotton product with broad application prospects [6,7,8,9,10]. Elucidating the molecular mechanism of pigment deposition and thus breeding more excellent NCC germplasm resources by genetic engineering and gene editing technologies have become major concerns [3,4,11,12,13,14].

Naturally brown cotton (NBC) is a major cultivar of NCC and holds significant importance in production applications. At the genetic level, studies have demonstrated that at least six loci (*Lc1*–*Lc6*) are associated with brown fibers, of which *Lc1* and *Lc2* produce brown fibers, *Lc3* is related to the dark brown fiber color, and *Lc4*, *Lc5*, and *Lc6* are responsible for producing light brown fiber [15,16]. Lc1 encodes an R2R3-type MYB transcription factor (TF) that regulates the expression of flavonoid biosynthesis pathway genes [14,17]. The genes distributed in the flavonoid biosynthesis pathway, such as *chalcone synthase* (*CHS*), *flavanone 3-hydroxylase* (*F3H*), *flavonoid 3′,5′-hydroxylase* (*F3′5′H*), *chalcone isomerase* (*CHI*), *leucoanthocyanidin reductase* (*LAR*), *dihydroflavonol 4-reductase* (*DFR*), *anthocyanidin reductase* (*ANR*), *flavonoid 3-O-glucosyltransferase* (*UFGT*), and *anthocyanidin synthase* (*ANS*), are closely associated with pigment synthesis in brown cotton fibers (BCFs) [13,14,18,19,20,21,22]. Silencing the *CHI* gene expression in brown cotton resulted in three fiber color phenotypes of light brown, green, and almost white, while overexpression of *3-O-Glucosyltransferase* (*3GGT*) in green cotton lines led to the formation of brown fibers [1]. *LAR* is a key structural gene in catalyzing the biosynthesis of Proanthocyanidins (PAs), and the transgenic results of *LAR* and *ANR* contribute to the development of new colored cotton germplasm [23].

In terms of secondary metabolites, evidence has indicated that the color of BCFs may be influenced by flavonoids [1,14,24,25]. Flavonoids constitute the largest class of secondary metabolites and are major components of plant pigments [1,12,26,27,28]. PAs have been confirmed to be a significant component of the pigments in BCFs [3,29]. Other compounds such as Leucodelphinidin, Leucocyanidin, Kaempferol, Epiafzelechin, Epicatechin, and (−)-Epigallocatechin are significantly accumulated in BCFs [14].

Although existing research on the pigment synthesis genes and metabolites in BCFs has been reported, the regulatory mechanism underlying the pigment deposition is still relatively unclear. In this study, a conjoint analysis of the transcriptomes and metabolomes of BCFs compared to white fibers at different developmental periods (0, 5, 10, 15, and 20 days post-anthesis, DPA) was performed to identify the regulatory genes and key pathways associated with pigment deposition. Our results showed that the differentially expressed genes, enriched pathways, and significantly accumulated metabolites located in the flavonoid biosynthesis pathway were identified, indicating the potentially significant roles of MYB-regulated expressions of *PAL*, *CHS*, *F3H*, *DFR*, *ANR*, and *UFGT* genes and of content accumulation of (−)-Epigallocatechin, Cyanidin-3-O-glucoside, Gallocatechin, and Myricetin in pigment deposition of BCFs. These results provide new insights for further understanding of the molecular mechanism of pigment synthesis and guidance for molecular design and breeding of excellent brown cotton materials.

## 2. Results

### 2.1. Comparative Transcriptomic Analysis between White and Brown Fibers

**Figure 1:**
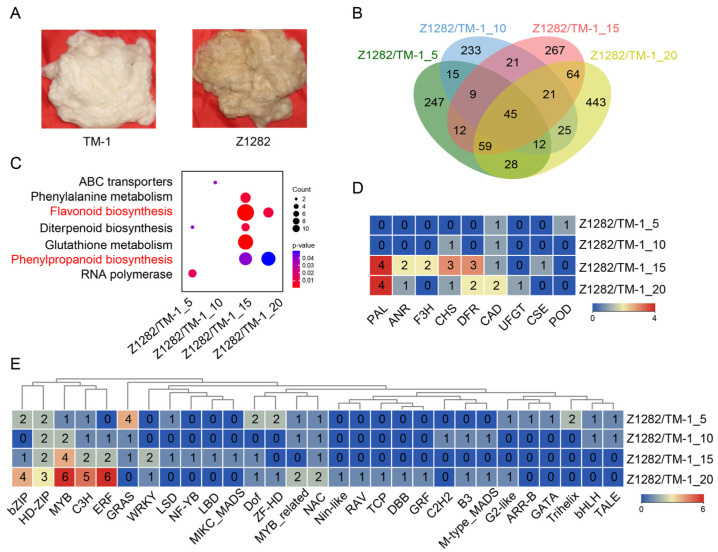
Comparative transcriptomic analysis between white (TM-1) and brown fibers (Z1282). (**A**) The mature fiber phenotypes of brown cotton Z1282 and white cotton TM-1. (**B**) Venn diagram of the UDEGs in Z1282 relative to TM-1 in 5, 10, 15, and 20 DPA fibers. (**C**) KEGG enrichment analysis of the UDEGs in 5, 10, 15, and 20 DPA fibers. (**D**) Statistical analysis of the number of UDEGs corresponding to different family members related to flavonoid biosynthesis and phenylpropanoid biosynthesis pathways. (**E**) Statistical analysis of the number of UDEGs corresponding to different family members of TFs. The clustering analysis was performed using hierarchical clustering by Euclidean distance and the complete linkage method.

We selected white (TM-1) and brown fibers (NBC Z1282) (Figure 1A) for transcriptomic sequencing at five periods of fiber development (0, 5, 10, 15, and 20 DPA) and successive comparative analysis. Under the screen criteria of |Log2(Fold change)| ≥ 1 and false discovery rate (FDR) < 0.05, compared to the corresponding periods for white fibers, a total of 427, 381, 498, and 697 up-regulated differentially expressed genes (UDEGs) in brown fibers at 5, 10, 15, and 20 DPA were identified, respectively (Figure 1B). We used Venn diagrams to illustrate the distribution of these UDEGs, revealing a total of 2003 UDEGs across the four periods and of 45 co-expressed UDEGs (CUDEGs) in all four periods (Figure 1B). KEGG analysis of these UDEGs at each stage indicated that seven pathways were enriched; interestingly, flavonoid biosynthesis and phenylpropanoid biosynthesis were the most significantly enriched pathways in the late periods of fiber development at 15 and 20 DPA (Figure 1C). Subsequently, we analyzed the genes involved in these two pathways, revealing a total of nine types of genes, including *phenylalanine ammonia lyase* (*PAL*), *ANR*, *F3H*, *CHS*, *DFR*, *Cinnamyl Alcohol Dehydrogenase* (*CAD*), *UFGT*, *Cinnamate-4-Hydroxylase* (*C4H*), and *Peroxidase* (*POD*) (Figure 1D). In the phenylpropanoid biosynthesis pathway, the enriched genes were mainly located at the beginning and end of the pathway (Figure S1), with PAL directly related to the initiation of this pathway and also associated with the flavonoid biosynthesis pathway. In the flavonoid biosynthesis pathway, *ANR*, *F3H*, *CHS*, and *DFR* were distributed at key positions within the pathway (Figure S2). These genes are likely key structural genes involved in pigment synthesis. Regarding the important role of TFs in regulating the expressions of genes distributed in the pathways of phenylpropanoid and flavonoid biosynthesis [14,17], we analyzed the TFs in these UDEGs, and the results showed that a total of 29 different types were discovered, of which 5 types indicated the most prominent enrichment, namely MYB, ERF, C3H, bZIP, and HD-ZIP, with over four members in each type. The majority of these TFs were significantly accumulated in the 15 and 20 DPA brown fibers. Specifically, MYB/MYB_related TFs had 13 members, suggesting their important potential roles as key regulatory genes (Figure 1E). These results indicate that both TFs, especially MYBs and UDEGs, related to the biosynthesis pathways of phenylpropanoids and flavonoids may act as crucial regulators for pigment synthesis.

### 2.2. Transcriptomic Analysis of Brown Fibers at Different Development Periods

**Figure 2:**
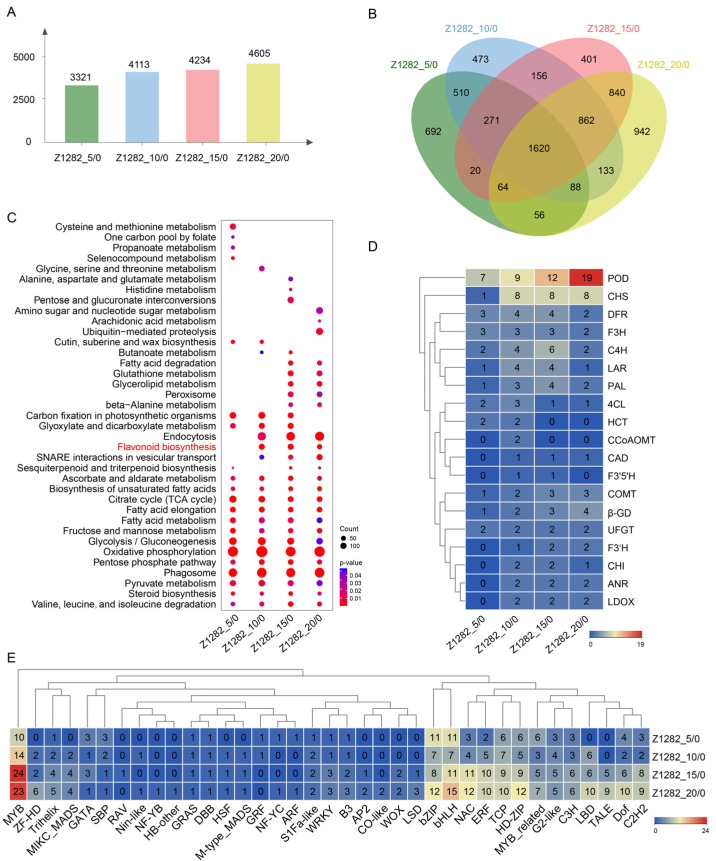
Transcriptomic analysis of different development periods of Z1282 fibers. (**A**) Statistical analysis of the number of UDEGs at 5, 10, 15, and 20 DPA relative to 0 DPA in Z1282 fibers. (**B**) Venn diagram of the UDEGs at 5, 10, 15, and 20 DPA relative to 0 DPA in Z1282 fibers. (**C**) KEGG enrichment analysis of the UDEGs in 5, 10, 15, and 20 DPA Z1282 fibers. (**D**) Statistical analysis of the number of UDEGs corresponding to different family members related to flavonoid biosynthesis and phenylpropanoid biosynthesis pathways. (**E**) Statistical analysis of the number of UDEGs corresponding to different family members of TFs. The clustering analysis was performed using hierarchical clustering by Euclidean distance and the complete linkage method.

A total of 3321, 4113, 4234, and 4605 UDEGs were identified in various periods of BCF development (5, 10, 15, and 20 DPA) compared to 0 DPA, respectively (Figure 2A). The Venn diagrams show that 7128 and 1620 were identified as UDEGs and CUDEGs in the four periods, respectively (Figure 2B). KEGG analysis indicated that 13 pathways were significantly enriched throughout the four periods, in which oxidative phosphorylation, phagosome, glycolysis/gluconeogenesis, and fatty acid-related pathways were prominent pathways (Figure 2C), indicating their potential involvement in BCF development. Interestingly, the flavonoid biosynthesis pathway was also observed at the periods of 10, 15, and 20 DPA.

Further analysis revealed that over 10 types of UDEGs were involved in this pathway, including *CHS*, *DFR*, *F3H*, *LAR*, *Hydroxycinnamoyltransferase* (*HCT*), *F3′5′H*, *UFGT*, *flavonoid 3′-hydroxylase* (*F3′H*), *CHI*, *ANR*, and *leucoanthocyanidin dioxygenase* (*LDOX*). These genes are distributed at key positions in the flavonoid biosynthesis pathway (Figure S4). Although the phenylpropanoid biosynthesis pathway was not significantly enriched, the genes in this pathway, such as *PAL*, *C4H*, and *4-coumarate: CoA ligase* (*4CL*), were also significantly up-regulated in all four periods of BCF development. Additionally, the genes located in related pathways, such as *POD*, *Caffeoyl-coenzyme A O-methyltransferase* (*CCoAOMT*), *Catechol-O-Methyltransferase* (*COMT*), *Cinnamyl alcohol dehydrogenase* (*CAD*), and *β-Glucuronidase* (*β-GD*), also indicated significantly up-regulated expression levels (Figure 2D) and may play crucial roles in regulating phenylpropanoid biosynthesis and metabolism (Figure S3). TF analysis displayed that a total of 37 different types of TFs were discovered, showing MYB/MYB_related, bHLH, NAC, bZIP, HD-ZIP, ERF, and TCP as the most abundant types. Remarkably, most of these TFs were predominantly accumulated in the late periods of 15 and 20 DPA, with MYB/MYB_related TFs being the most significantly enriched members (Figure 2E). These results suggest a possible close link between MYB and flavonoid biosynthesis pathway genes, which may jointly regulate brown fiber development.

### 2.3. Comprehensive Transcriptomic Analysis of Brown/White Fibers and Brown Fiber Development

To accurately locate and identify the key genes involved in pigment synthesis of brown fibers, we conducted a comprehensive analysis of the UDEGs that are specifically expressed in brown fibers compared to white fibers and expressed in a widespread manner in different periods (5, 10, 15, and 20 DPA) of brown fiber development compared to 0 DPA.

**Figure 3:**
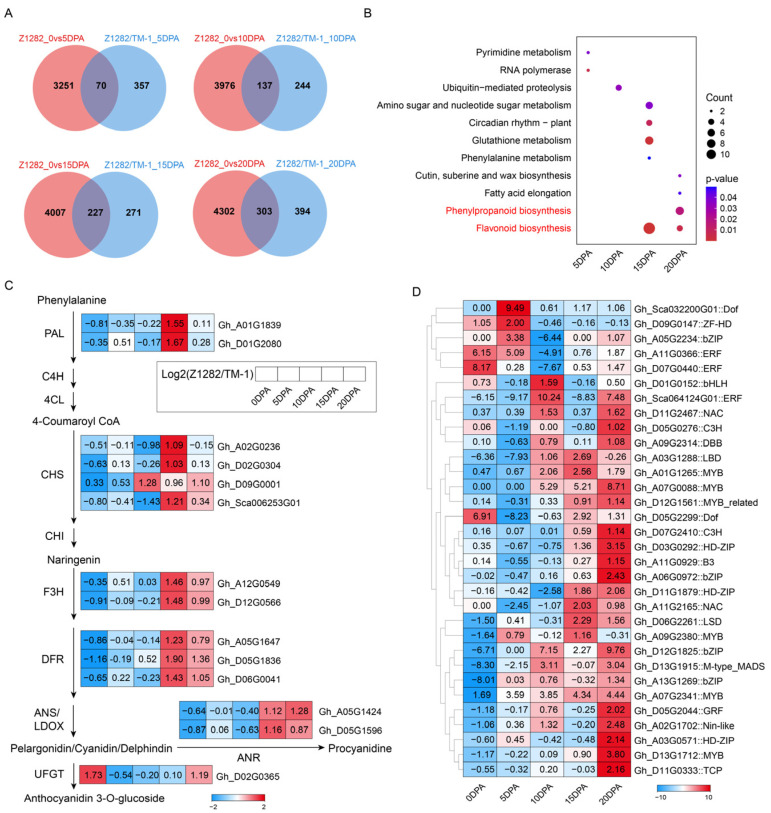
Multidimensional transcriptomic analysis of different development periods of Z1282 and TM-1 fibers. (**A**) Venn diagram of the UDEGs at 5, 10, 15, and 20 DPA in Z1282 and TM-1 fibers. (**B**) KEGG enrichment analysis of co-expressed UDEGs at 5, 10, 15, and 20 DPA in Z1282 and TM-1 fibers. (**C**) Heatmap of the expression levels of genes located in the pathways of flavonoid biosynthesis and phenylalanine biosynthesis in Z1282 relative to TM-1 at different fiber development periods. (**D**) Heatmap of the TF expression levels of Z1282 relative to TM-1 at different fiber development periods. The clustering analysis was performed using hierarchical clustering by Euclidean distance and the complete linkage method.

**Figure 4:**
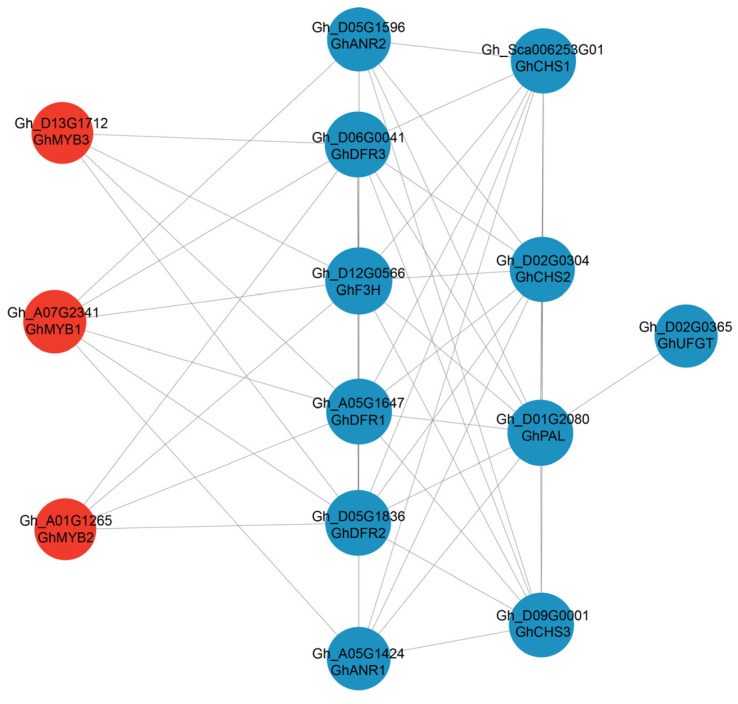
Protein–protein interaction (PPI) analysis of the co-expressed UDEGs located in the flavonoid biosynthesis pathway in different development periods for Z1282 and TM-1 fibers. Red and blue colors represent transcription factors and flavonoid biosynthesis pathway genes, respectively.

The results showed that there were 70, 137, 227, and 303 co-expressed UDEGs in the two dimensions, respectively (Figure 3A). KEGG analysis of these co-expressed UDEGs indicated that the pathways of flavonoid biosynthesis and phenylpropanoid biosynthesis were significantly enriched in the late periods of 15 and 20 DPA (Figure 3B). Further analysis demonstrated that these two pathways contained several types of genes, including *PAL*, *CHS*, *F3H*, *DFR*, *ANR*, and *UFGT*, which were more accumulated in brown fibers than in white fibers at 15 and 20 DPA (Figure 3C). Similarly, TFs also indicated significantly enriched expressions in these two periods of 15 and 20 DPA brown fibers (Figure 3D). These genes are closely related to the synthesis of flavonoid metabolites. Protein–protein interaction (PPI) analysis results showed that three MYB TFs were involved in the regulation of these genes. Among them, Gh_D13G1712 (GhMYB3) and Gh_A01G1265 (GhMYB2) may directly participate in the regulation of *Gh_D06G0041* (*GhDFR3*), *Gh_A05G1647* (*GhDFR1*), *Gh_D05G1836* (*GhDFR2*), and *Gh_D12G0566* (*GhF3H*). Besides the regulation of the aforementioned four genes, Gh_A07G2341 (GhMYB1) also participates in the regulation of *Gh_A05G1424* (*GhANR1*) and *Gh_D05G1596* (*GhANR2*). These results suggest that the three MYB TFs may affect the synthesis of different flavonoid metabolites by regulating the six genes, thereby possibly controlling the pigment synthesis of brown fibers. Additionally, five genes—*Gh_Sca006253* (*GhCHS1*), *Gh_D02G0304* (*GhCHS2*), *Gh_D09G0001* (*GhCHS3*), *Gh_D01G2080* (*GhPAL*), and *Gh_D02G0365* (*GhUFGT*)—also indicated similar expression patterns to the abovementioned genes regardless of the non-direct regulation by the three TFs (Figure 4).

**Figure 5:**
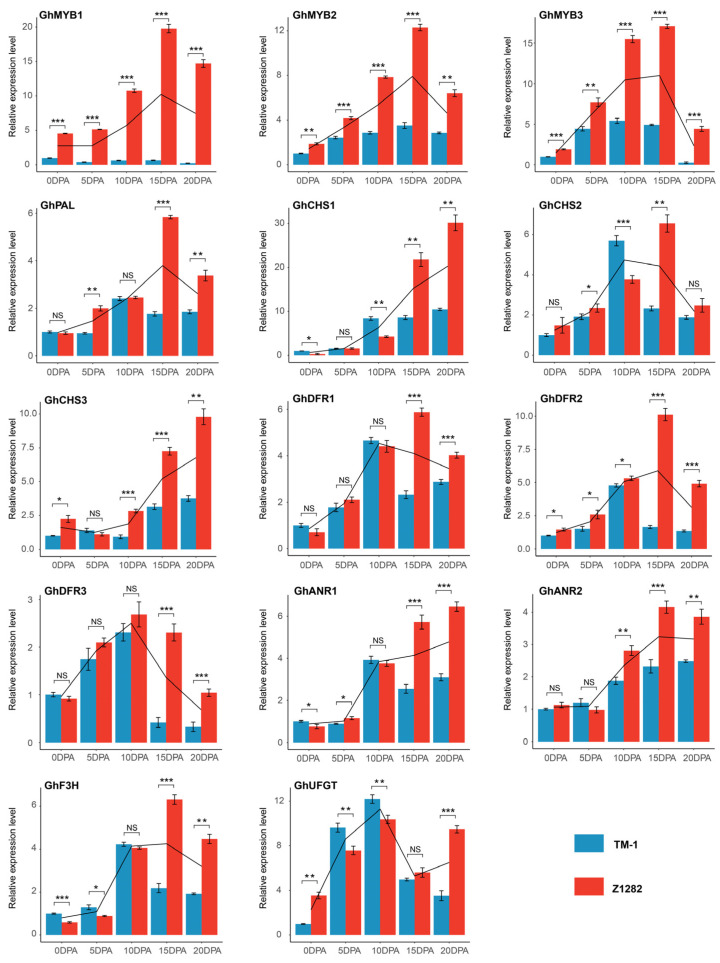
RT-qPCR validation of candidate transcription factors and flavonoid biosynthesis pathway genes. The genes of transcription factors (*GhMYB1-GhMYB3*) and flavonoid biosynthesis pathway genes (*GhPAL*, *GhCHS1-3*, *GhDFR1-3*, *GhANR1-2*, *GhF3H*, and *GhUFGT*) were selected for RT-qPCR detections using the materials of 0, 5, 10, 15, and 20 DPA ovules and fibers of Z1282 and TM-1 as templates. The black lines represent the trends of gene expression levels during different development periods for Z1282 and TM-1 fibers. The *t*-test was used for significant difference analysis, with *, **, and *** denoting significant differences at *p* < 0.05, 0.01, and 0.001 levels, respectively, and NS representing no significant difference.

To verify the reliability of the transcriptomic data, we performed RT-qPCR validation on the expression levels of three MYB genes and eleven flavonoid biosynthesis pathway genes, showing a high consistent expression tendency between the transcriptomes and RT-qPCR detections (Figure 5). These results suggest that three MYBs and nine flavonoid pathway genes are significantly accumulated in the late developmental periods of brown fibers and may be closely related to the synthesis and deposition of pigments in brown fibers.

### 2.4. Comparative Metabolomic Analysis between White and Brown Fibers

**Figure 6:**
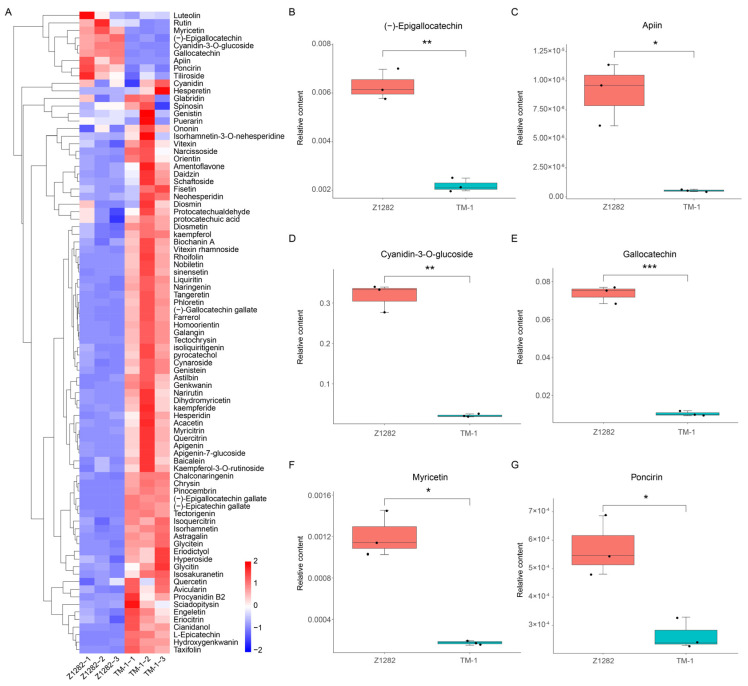
Analysis of flavonoid metabolite levels in Z1282 and TM-1 fibers through extensive targeted metabolomics. (**A**) Heatmap and clustering analysis of content levels of all detected flavonoid metabolites. The clustering analysis was performed using hierarchical clustering by Euclidean distance and the complete linkage method. (**B**–**G**) Box plots of six significantly up-regulated metabolites in fibers of Z1282 relative to TM-1. *, **, and *** denote significant differences at *p* < 0.05, 0.01, and 0.001 levels.

Considering the significantly enriched UDEGs and pathway for flavonoid biosynthesis, to further investigate the specific relationship between the UDEGs and metabolites, we performed an extensive targeted metabolomic analysis of flavonoid metabolites in brown and white cotton fibers. The results displayed that a total of 85 flavonoid metabolites were identified. Principal component analysis (PCA) showed a clear separation between white fibers and BCFs (Figure S5A). Analyses of a volcano plot of hierarchical clustering and Orthogonal Projections to Latent Structures Discriminant Analysis (OPLS-DA) of metabolite relative quantification values showed that nine metabolites were significantly accumulated in brown fibers (Figure 6A and Figure S5B). Further analysis indicated that six metabolites, (−)-Epigallocatechin, Apiin, Cyanidin-3-O-glucoside, Gallocatechin, Myricetin, and Poncirin, were significantly up-regulated in brown fibers, with low levels in white fibers (Figure 6B–G), while in terms of the content of the other three metabolites, Luteolin, Tiliroside, and Rutin, no significant differences were detected between white and brown fibers (Figure S6). These results suggest that the six significantly enriched metabolites may be key substances for pigment synthesis in brown fibers.

### 2.5. Conjoint Analysis of Transcriptomes and Metabolomes of Brown and White Fibers

**Figure 7:**
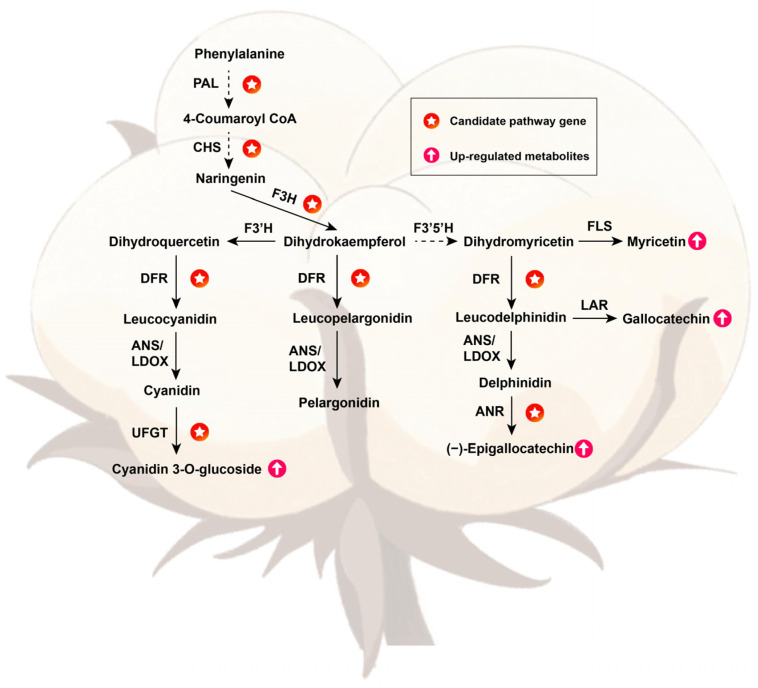
Schematic diagram of candidate genes and metabolites in flavonoid biosynthesis and phenylalanine biosynthesis pathways. The pathways were constructed based on the KEGG flavonoid and phenylalanine biosynthesis pathways. Red stars and pink arrows represent the candidate UDEGs and up-regulated metabolites in Z1282 fibers.

To further investigate the correlation between the UDEGs and the metabolites, we performed a comprehensive conjoint analysis of the UDEGs and metabolites involved in the flavonoid biosynthesis pathway, and constructed a pathway model diagram. It appeared that (−)-Epigallocatechin, Cyanidin-3-O-glucoside, Gallocatechin, and Myricetin are significantly accumulated and directly related to flavonoid biosynthesis. The UDEGs of *PAL*, *CHS*, and *F3H* are located in the upstream of these four metabolites and are involved in the synthesis of all these metabolites. *DFR* is distributed in the upstream of Gallocatechin, Cyanidin-3-O-glucoside, and (−)-Epigallocatechin to participate in the synthesis of the three metabolites. *FLS*, *ANR*, and *UFGT* are directly involved in the synthesis of Myricetin, (−)-Epigallocatechin, and Cyanidin-3-O-glucoside, respectively (Figure 7). These results suggest that the UDEGs may perform a direct or indirect function in catalyzing the synthesis of these metabolites and thus jointly affecting the pigment synthesis of brown fibers.

## 3. Discussion

### 3.1. Flavonoid Biosynthesis and Phenylpropanoid Biosynthesis Pathways Are Enriched during the Development of BCFs

Cotton fiber development goes through different periods, namely fiber initiation (0–3 DPA), primary wall synthesis and elongation (3–15 DPA), transition from primary to secondary walls (15–20 DPA), secondary wall synthesis (20–40 DPA), and fiber maturation (40–50 DPA) [30,31,32]. In addition to fiber development, NCCs also undergo the process of pigment synthesis and deposition [1]. In this study, by a comparative transcriptomic analysis of brown and white cotton fibers at the four periods of 5, 10, 15, and 20 DPA, we found that the UDEGs and enriched pathways were involved in flavonoid biosynthesis and phenylpropanoid biosynthesis pathways, particularly during the late developmental periods of 15 and 20 DPA for brown fibers (Figure 1C), showing consistency with previous studies [14]. Transcriptomic analysis of various developmental periods of brown fibers indicated that the flavonoid biosynthesis pathway was a significantly enriched pathway (Figure 2C). Anthocyanin synthesis in BCFs has been reported to lead to a shortened elongation period [33]. Genes related to the flavonoid biosynthesis pathway, *DFR*, *FLS*, and *ANS*, have been reported as key genes in the synthesis and accumulation of anthocyanins in *Populus* × *euramericana* cv. “Zhonghuahongye” [34]. *TgCHS*, *TgFLS*, *TgF3H*, *TgF3′H*, *TgF3′5′H*, and *TgDFR*, as key structural genes of the flavonoid biosynthesis pathway, are involved in anthocyanin synthesis during tulip flower development [35].

Combined analysis of the two dimensions of brown fiber development and comparison of white and brown fibers identified the flavonoid biosynthesis pathway as the most significantly enriched pathway; additionally, the phenylpropanoid biosynthesis pathway that serves as the precursor to the flavonoid biosynthesis pathway as well as the related UDEGs were also discovered (Figure 3B,C), suggesting the potentially important role of flavonoid metabolites in pigment synthesis of brown fibers. It has been reported that the flavonoid biosynthesis pathway is related to pigment synthesis in sunflower flowers [28]. The flavonoid biosynthesis pathway is a major pathway for pigment synthesis in plants [27].

### 3.2. Flavonoid Biosynthesis Pathway Genes and MYBs Are the Main Regulatory Genes during the Development of BCFs

Previous studies have shown that the synthesis and deposition of pigments in NCC fibers are significantly associated with flavonoid biosynthesis pathway genes [1,4,14]. Silencing the *GhCHI1* gene in brown cotton resulted in three color phenotypes: brown, green, and white. Overexpression of *Gh3GT* and *At3GT* led to the transition of brown to green fibers [1]. *GhANR1*, *GhANR2*, and *GhANS* showed significantly increased transcriptomic and proteomic levels at 15 DPA in brown fibers compared to white fibers [14]. *GhUFGT1/2* are expressed in brown fibers from 1 to 30 DPA, with the highest expression at 25 and 30 DPA [22]. In our study, the flavonoid biosynthesis and phenylpropanoid biosynthesis pathways were significantly enriched during pigment synthesis and deposition in brown cotton (Figure 2C), and the genes distributed in the flavonoid and phenylpropanoid pathways were also prominently up-regulated (Figure 2D), which is consistent with previous research results in which almost all these genes were highly enriched in brown fibers [14,29]. After a combined comparative transcriptomic analysis of brown and white fibers, we further identified the flavonoid pathway-related genes, including *PAL*, *CHS*, *F3H*, *DFR*, *ANR*, and *UFGT* (Figure 3C). A total of 5 MYBs and 1 MYB_related TF out of 32 TFs were discovered as predominantly accumulated genes in brown fibers (Figure 3D), and further PPI analysis indicated that three MYBs (GhMYB1–GhMYB3) have possible direct regulatory relationships with the pathway genes of *GhANR*, *GhDFR*, and *GhF3H* (Figure 4). Reports have shown that MYBs are closely associated with pigment synthesis in brown cotton [17]. The gene *Lc1*, which encodes an MYB, can regulate the expressions of flavonoid pathway genes in brown cotton [15,16]. GhMYB3, GhMYB6, and GhMYB46 can bind to the promoters of *GhANS*, *GhANR1*, and *GhUFGT2* to regulate their expressions [14]. It is inferred that the three MYBs may be involved in pigment synthesis via regulating the expressions of flavonoid pathway genes.

### 3.3. Regulatory Network of Flavonoid Pathway Genes and Metabolites Related to Pigment Synthesis and Deposition

**Figure 8:**
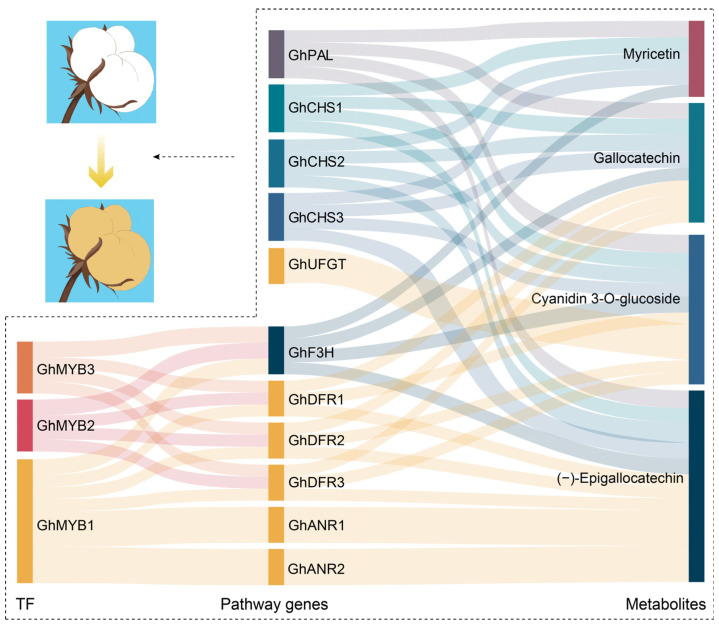
Proposed schematic diagram of regulatory network of TFs and flavonoid biosynthesis pathway genes and metabolites involved in pigment deposition of brown cotton fibers. The connections between transcription factors and flavonoid biosynthesis pathway genes denote the transcriptomic regulatory interactions. The connections between flavonoid biosynthesis pathway genes and metabolites represent the possibility that these genes directly participate in the synthesis of corresponding metabolites.

Evidence has indicated that PAs are important components of BCFs. Our comparative targeted metabolomic analysis of flavonoid metabolites in brown and white cotton fibers demonstrated that the contents of the six components (−)-epigallocatechin, apiin, cyanidin-3-O-glucoside, gallocatechin, myricetin, and poncirin are significantly higher in BCFs (Figure 6). Meanwhile, by a conjoint analysis of the UDEGs and metabolites of the flavonoid pathway, we further identified three MYBs (GhMYB1–GhMYB3), eleven pathway genes (*GhANR1*–*GhANR2*, *GhF3H*, *GhDFR1*–*GhDFR3*, *GhCHS1*–*CHS3*, *GhPAL*, and *GhUFGT*), and four metabolites [(−)-epigallocatechin, cyanidin-3-O-glucoside, gallocatechin, and myricetin] to construct a regulatory network for the synthesis and deposition of pigments in BCFs (Figure 8). Flavonoid pathway metabolites such as leucodelphinidin, leucocyanidin, kaempferol, epiafzelechin, epicatechin, and epigallocatechin have higher levels in BCFs [3,13,14], suggesting the important function of these metabolites in regulating the pigment deposition of brown fibers. The molecular interactions of MYB and flavonoid pathway genes and the genetic validation of these genes in brown cotton are expected to be confirmed in the future.

## 4. Materials and Methods

### 4.1. Plant Materials

Brown cotton cultivar Zong1282 (Z1282) and TM-1 were planted in the experimental fields of Shihezi University. Ovules and fibers were collected at 0, 5, 10, 15, and 20 DPA and stored at −80 °C before use.

### 4.2. RNA Extraction, cDNA Library Construction, and Sequencing

RNA was extracted from 30 independent samples across five developmental periods (0, 5, 10, 15, and 20 DPA) of Z1282 and TM-1 using the RNAprep Pure Plant Plus Kit (Code No. DP441; Tiangen, Beijing, China) following the manufacturer’s protocol. The RNA quantity was measured with a NanoDrop ND2000 spectrophotometer (NanoDrop Technologies, Wilmington, DE, USA). RNA quality was assessed with an Agilent Bioanalyzer 2100 system (Agilent Technologies, Palo Alto, CA, USA). RNA integrity was confirmed by 1% agarose gel electrophoresis. High-quality RNA was used to prepare RNA-seq libraries, with three biological replicates for each sample. Transcriptome sequencing was performed on the HiSeq 2000 Sequencing System (Illumina, San Diego, CA, USA).

### 4.3. RNA Sequencing Data Analysis

Raw data were quality-controlled using Trimmomatic to remove adapters and low-quality sequences with the parameters “ILLUMINACLIP:TruSeq3-PE.fa:2:30:10 LEADING:3 TRAILING:3 SLIDINGWINDOW:4:15 MINLEN:50” [36]. The base quality of the trimmed reads was checked using FastQC (Burks and Azad, 2022) [37]. Clean reads were aligned to the Gossypium hirsutum TM-1 genome (CRI v1) using Hisat2 [38]. The reference genome was obtained from the Cotton Functional Genomics Database (CottonFGD, https://cottonfgd.net/about/download.html, accessed on 20 March 2024) [39]. Gene expression levels were calculated using Stringtie (Version 1.3.3b) software and quantified as fragments per kilobase of transcript per million mapped reads (FPKM) [40]. Differentially expressed genes (DEGs) were identified using the R package DESeq2. The resulting *p*-values were adjusted for FDR using the Benjamini and Hochberg method [41]. Genes conforming to the screen conditions of |Log2(Fold change)| ≥ 1 and *FDR* < 0.05 were considered as DEGs [1].

### 4.4. TF Prediction

For the screened DEGs, protein sequences were batch-extracted using the Tbtools-Fasta Extract module based on gene IDs and then submitted to the Plant TF Database (PlantTFDB v5.0, https://planttfdb.gao-lab.org/prediction.php, accessed on 10 May 2024) for prediction [42,43].

### 4.5. KEGG Enrichment and PPI Analysis of DEGs

Kyoto Encyclopedia of Genes and Genomes (KEGG) pathway enrichment analysis of DEGs was conducted using the KEGG Orthology-Based Annotation System (KOBAS3.0, http://bioinfo.org/kobas/genelist/, accessed on 18 May 2024) [44]. Protein–protein interaction network (PPI) analysis was performed using the STRING database (https://cn.string-db.org/, accessed on 28 May 2024) [45]. Both KEGG and PPI analyses used protein sequences of DEGs as input sequences, selecting *Gossypium hirsutum* as the organism.

### 4.6. Real-Time Quantitative Polymerase Chain Reaction (RT-qPCR) Analysis

RT-qPCR was performed using the templates same with the RNA samples for transcriptome sequencing. First-strand cDNA synthesis was carried out using the FastKing RT Kit (Code No. KR116; Tiangen, Beijing, China) according to the manufacturer’s instructions. Subsequently, RT-qPCR was conducted using Novostar SYBR qPCR SuperMix Plus (Code No. E096-01A; Novoprotein, Shanghai, China) on a LightCycler 480 (Roche, Mannheim, Germany). All data were normalized to the expression level of GhUBQ7 [1]. Specific primers were designed using the Primer-BLAST tool (http://www.ncbi.nlm.nih.gov/tools/primer-blast, accessed on 10 June 2024) [46].

### 4.7. Metabolomic Analysis of Cotton Fibers

Fibers of Z1282 and TM-1 were naturally dried for 30 days in a laboratory’s constant temperature room, followed by metabolite extraction and identification and quantification of metabolites, following the method described by Liu et al. [1]. Variable importance in the projection (VIP) of partial least-squares discriminant analysis (PLS-DA) was used to display changes in metabolites among different samples [47]. The selection criteria for differential metabolites were as follows: (1) VIP ≥ 1, (2) fold change ≥ 1.2, (3) *q*-value < 0.05 [14]. The Euclidean distance matrix of relative metabolite quantification values was calculated and clustered using the complete linkage method, performed with the Tbtools-Heatmap module [43]. For candidate metabolites, quantitative results were displayed using box plots, and significant analysis of metabolites was conducted using Student’s *t*-test [48]. Significant differences were represented with one to three asterisks for *p* < 0.05, *p* < 0.01, and *p* < 0.001, respectively.

## 5. Conclusions

Through multidimensional transcriptomic, metabolic, and conjoint analyses of brown and white fibers, we found that the UDEGs, enriched pathways, and significantly accumulated metabolites associated with biosynthesis were the key factors for pigment synthesis and deposition in brown fibers. Based on these flavonoid pathway components, we also constructed a regulatory network. Our results provide solid foundations and effective candidates for elucidation of the mechanism of pigment deposition and for breeding of excellent colored cotton materials.

## Data Availability

All sequencing data generated for this work have been deposited in the NCBI Gene Expression Omnibus (GEO) under accession number GSE76400. All authors agree with the MDPI Research Data Policies.

## Supplementary Materials

The following supporting information can be downloaded at https://www.mdpi.com/article/10.3390/plants13152028/s1. Figure S1: Pathway diagram of DEGs in the phenylalanine biosynthesis pathway of Z1282 vs. TM-1 transcriptome. Figure S2: Pathway diagram of DEGs in the flavonoid biosynthesis pathway of Z1282 vs. TM-1 transcriptome. Figure S3: Pathway diagram of DEGs in the phenylalanine biosynthesis pathway of Z1282 fiber development transcriptome. Figure S4: Pathway diagram of DEGs in the flavonoid biosynthesis pathway of Z1282 fiber development transcriptome. Figure S5: Overview of metabolite samples. Figure S6: Box plots of other three up-regulated metabolites in Z1282 relative to TM-1. Table S1: List of RT-qPCR primers used in this study.
