# Supplementary material for: Integrative Transcriptomic and Metabolic Analyses Reveal That Flavonoid Biosynthesis Is the Key Pathway Regulating Pigment Deposition in Naturally Brown Cotton Fibers
Source: Plants (Basel). 2024 Jul 24;13(15):2028. doi: 10.3390/plants13152028 (PMC11314106; doi:10.3390/plants13152028)
Supplement: Supplementary file 1 [file plants-13-02028-s001.zip › plants-3082222-supplementary.pdf]

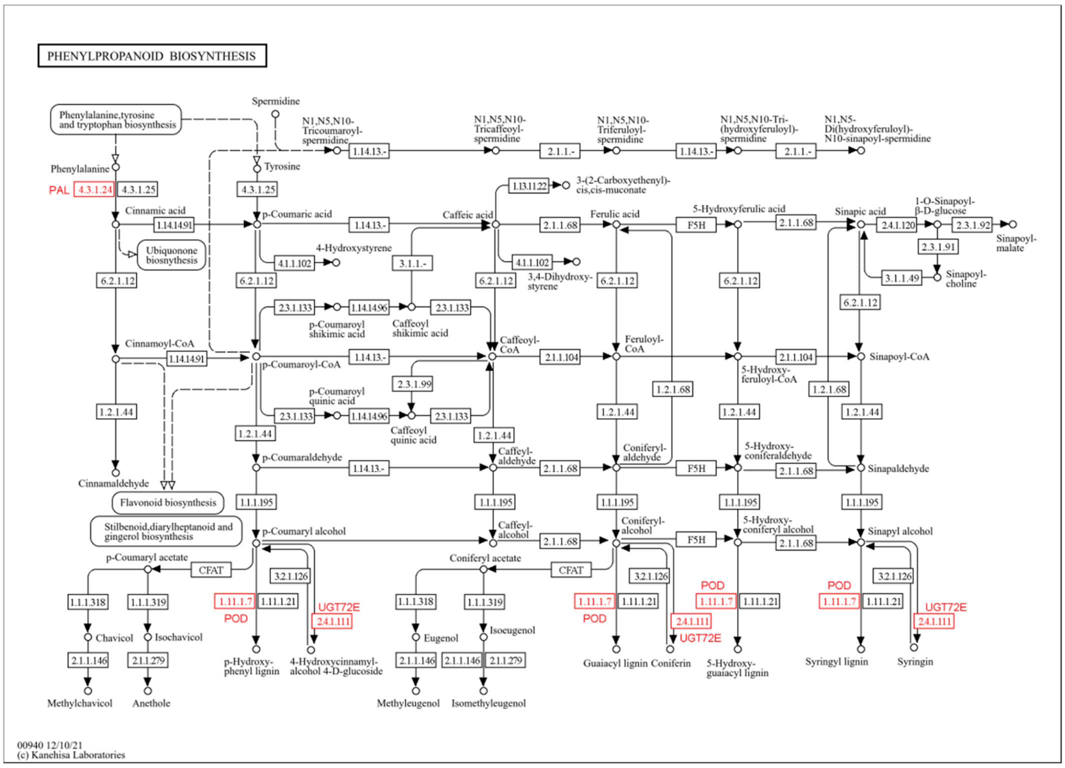

Figure S1. Pathway diagram of DEGs in the phenylalanine biosynthesis pathway of Z1282 vs TM-1 transcriptome. Red indicates up-regulated genes.

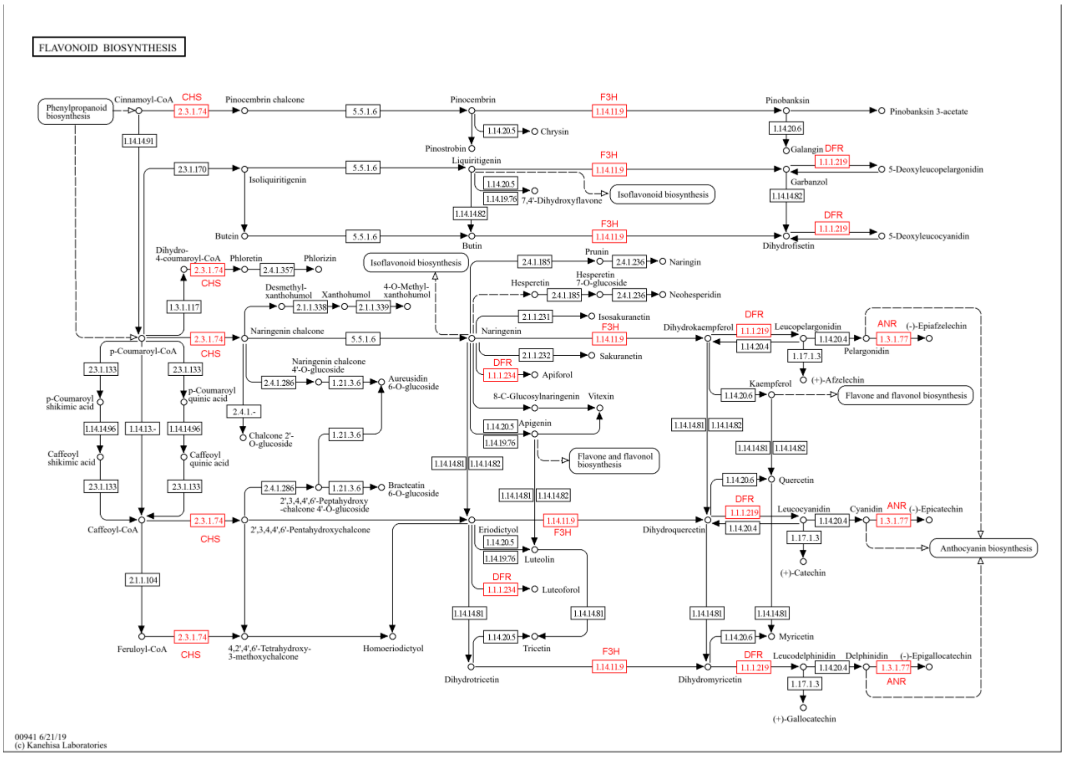

Figure S2. Pathway diagram of DEGs in the flavonoid biosynthesis pathway of Z1282 vs TM-1 transcriptome. Red indicates up-regulated genes.

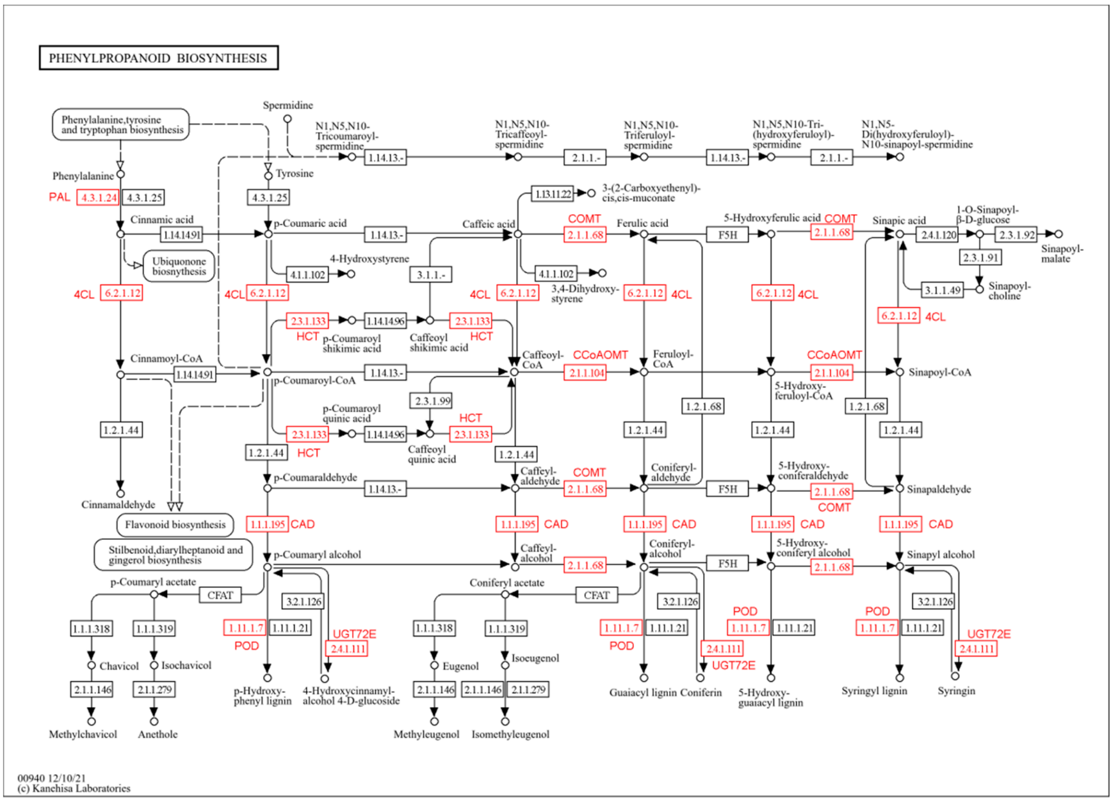

Figure S3. Pathway diagram of DEGs in the phenylalanine biosynthesis pathway of Z1282 fiber development transcriptome. Red indicates up-regulated genes.

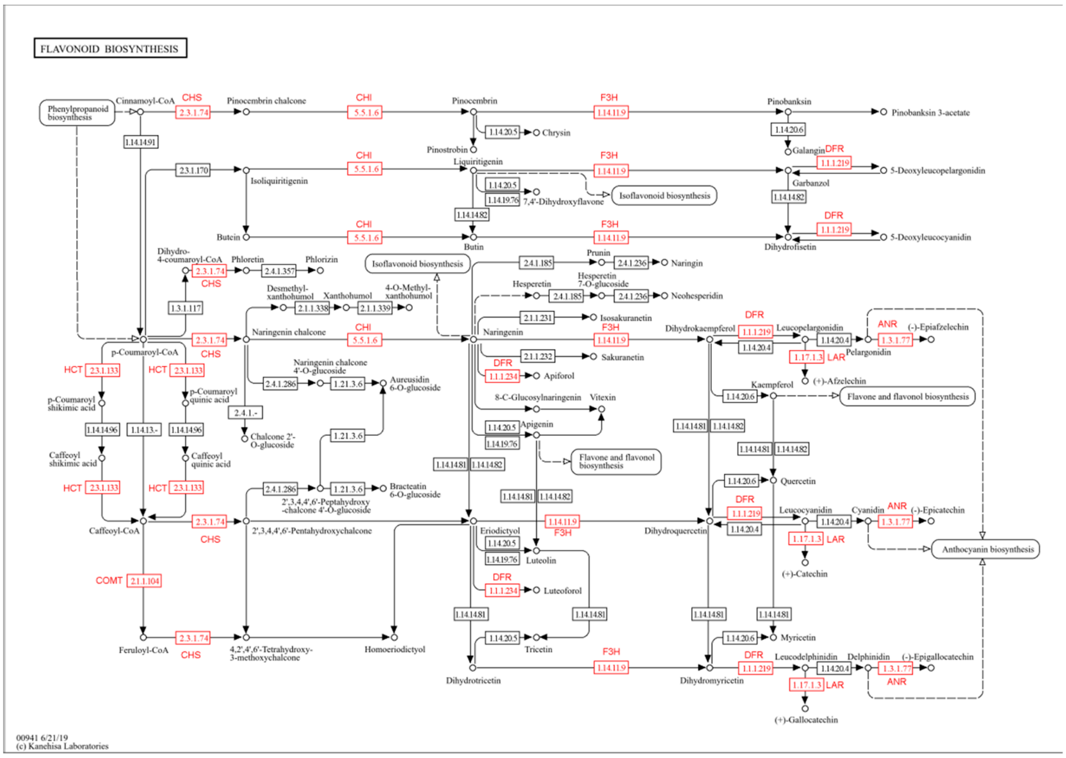

Figure S4. Pathway diagram of DEGs in the flavonoid biosynthesis pathway of Z1282 fiber development transcriptome. Red indicates up-regulated genes.

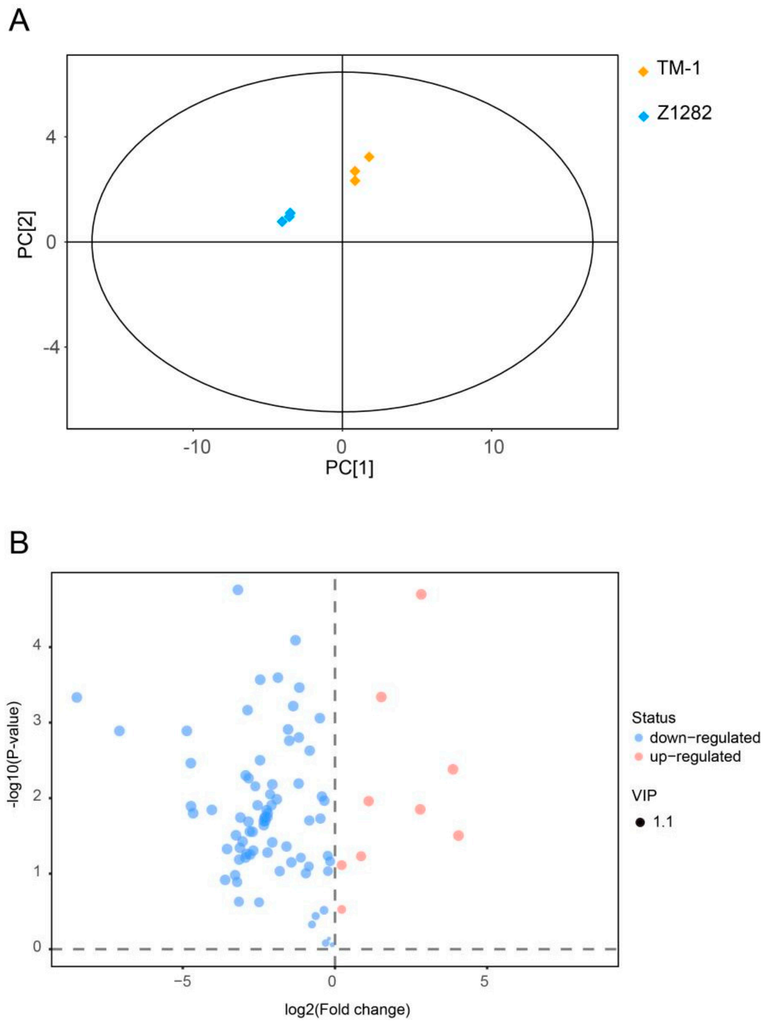

Figure S5. Overview of metabolite samples.

(**A**) PCA analysis of metabolic samples from TM-1 and Z1282. (**B**) The differential changes of all flavonoid metabolites.

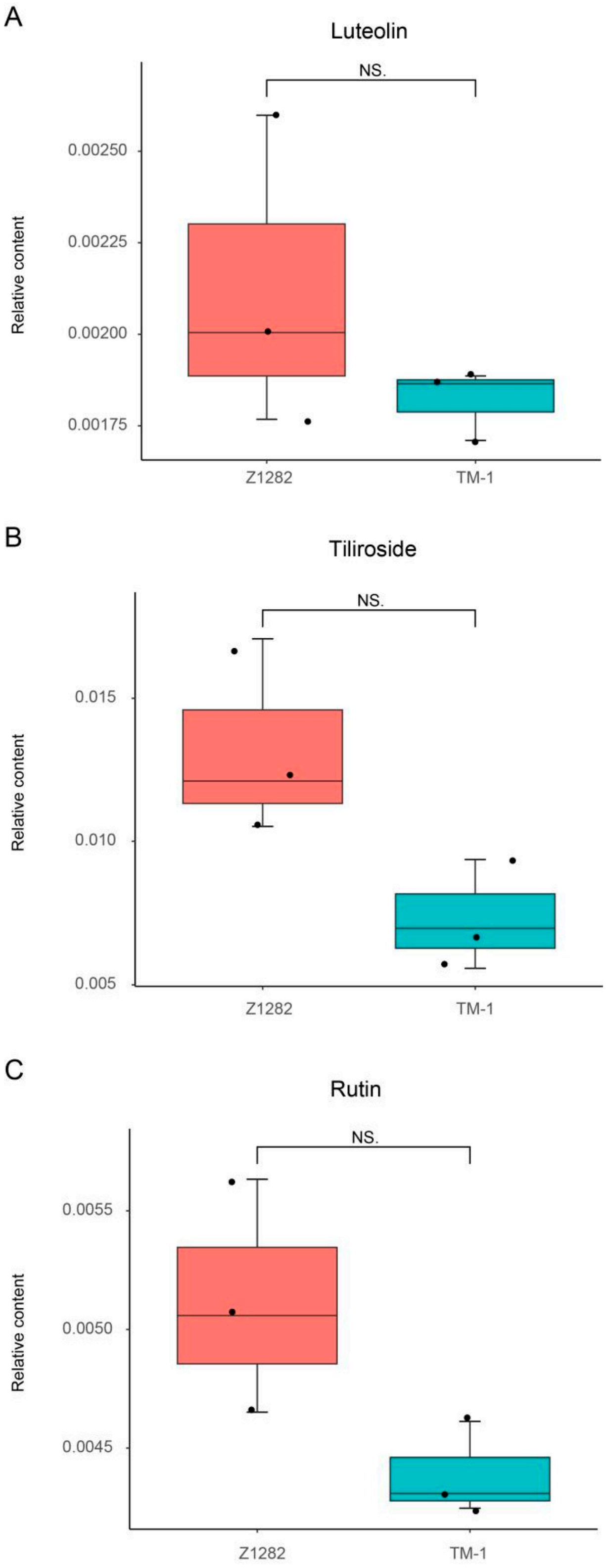

Figure S6. Box plots of other three up-regulated metabolites in Z1282 relative to TM-1.

Table S1. List of RT-qPCR primers used in this study.

Gene name	Sequence (5' - 3')
qGhANR1-F	CAAGTTGTTGCTCGAGAAGG
qGhANR1-R	CTTCTTCTGGTTGTCAGGGT
qGhANR2-F	TCAACACACTGAATGGGACA
qGhANR2-R	TGGTGGCTTTGCTGATGATA
qGhCHS1-F	TTGTTTGCTCGGAAATCACC
qGhCHS1-R	TTGTTTGCTCGGAAATCACC
qGhCHS2-F	CACTAAGCTTTTGGGCCTTC
qGhCHS2-R	TTGATACATCATGAGGCGCT
qGhCHS3-F	CGGCCTCAATTTCCATTTGT
qGhCHS3-R	ATGCCAATTGGTGAGAATGC
qGhDFR1-F	GAATCCAAAAGCGGAAGGTC
qGhDFR1-R	AATGGTAGCATGGTGAGAGG
qGhDFR2-F	GAATCCAAAAGCGGAAGGTC
qGhDFR2-R	AATGGTAGCATGGTGAGAGG
qGhDFR3-F	TGGAAGACATGTTTGTCGGA
qGhDFR3-R	TGGAGGTGCAATTAACCCTT
qGhF3H1-F	ACACTCTTGCTTCAAGACCA
qGhF3H1-R	TTCTTGAACCTCCCATTGCT
qGhPAL1-F	GAACACGGTGAAGAACACTG
qGhPAL1-R	AATCTCGATGGGTGGAGTTC
qGhUGFT1-F	TGTTGGAATTAGCTTGGGGT
qGhUGFT1-R	CTATAGGCGGACGAACTACC
qGhMYB1-F	GGGCATCGATCCAAATAAGC
qGhMYB1-R	AATGTTGCACTTGCACTTGT
qGhMYB2-F	TCGACATGGTCAACATCACT
qGhMYB2-R	GCAATACCACCACCCATTTC
qGhMYB3-F	AGAAAGTGAGACTGGAGCTG
qGhMYB3-R	GTGACGATCTCATCCAACCT
UBQ7-F	AAGAAGAAGACCTACACCAAGCC
UBQ7-R	GCCCACACTTACCGCAATA
